# High Sensitivity of Aged Mice to Deoxynivalenol (Vomitoxin)-Induced Anorexia Corresponds to Elevated Proinflammatory Cytokine and Satiety Hormone Responses

**DOI:** 10.3390/toxins7104199

**Published:** 2015-10-19

**Authors:** Erica S. Clark, Brenna M. Flannery, Elizabeth M. Gardner, James J. Pestka

**Affiliations:** 1 Department of Food Science and Human Nutrition, Michigan State University, East Lansing, MI 48824, USA; E-Mails: clarke27@anr.msu.edu (E.S.C.); bflannery27@gmail.com (B.M.F.); egardner@anr.msu.edu (E.M.G.); 2 Center for Integrative Toxicology, Michigan State University, East Lansing, MI 48824, USA; 3 Department of Microbiology and Molecular Genetics, Michigan State University, East Lansing, MI 48824, USA

**Keywords:** mycotoxin, anorexia, aging, weight loss, PYY, CCK, IL-6, IL-1β, mouse

## Abstract

Deoxynivalenol (DON), a trichothecene mycotoxin that commonly contaminates cereal grains, is a public health concern because of its adverse effects on the gastrointestinal and immune systems. The objective of this study was to compare effects of DON on anorectic responses in aged (22 mos) and adult (3 mos) mice. Aged mice showed increased feed refusal with both acute i.p. (1 mg/kg and 5 mg/kg) and dietary (1, 2.5, 10 ppm) DON exposure in comparison to adult mice. In addition to greater suppression of food intake from dietary DON exposure, aged mice also exhibited greater but transient body weight suppression. When aged mice were acutely exposed to 1 mg/kg bw DON i.p., aged mice displayed elevated DON and DON3GlcA tissue levels and delayed clearance in comparison with adult mice. Acute DON exposure also elicited higher proinflammatory cytokine and satiety hormone responses in the plasma of the aged group compared with the adult group. Increased susceptibility to DON-induced anorexia in aged mice relative to adult mice suggests that advanced life stage could be a critical component in accurate human risk assessments for DON and other trichothecenes.

## 1. Introduction

Deoxynivalenol (DON, vomitoxin), a trichothecene mycotoxin produced by the fungus *Fusarium graminearum*, is a common cereal grain contaminant that is highly resistant to heat processing, leading to contamination of human and animal food [1]. Adverse effects of acute exposure to DON include anorexia, diarrhea, and vomiting in experimental animals [2]. In addition, chronic DON exposure can lead to immunotoxic effects and growth retardation. Mice, commonly used in experimental studies for DON risk assessment, are incapable of vomiting but exhibit feed refusal and body weight suppression following exposure to the toxin [3].

Previous studies have investigated sex differences and the susceptibility of young animals to DON-induced anorexia [4,5,6,7,8]; however, the effects of this toxin on aged animals are largely unaddressed. Studying the adverse effects of DON in advanced life stage animals is important because approximately 25% and 30% of elderly men and women, respectively, are reported to be in an anorectic state [9,10,11]. This phenomenon of unintentional weight loss in advanced life stage has been termed the “anorexia of aging” and is a high predictor of morbidity and mortality in the elderly [12,13]. The etiology of anorexia is complex and thought to be caused by many factors including depression, increased susceptibility to illness, diet, and the burden of multiple medications [10,14,15]. In a study of elderly Finnish men with depressive symptoms (65–84 years of age; *n* = 688), many reported gastrointestinal complaints over a two-week period including diarrhea (20%), stomach pains (37%), nausea (29%), vomiting (9%), and loss of appetite (21%) [16]. As these symptoms are also present with DON intoxication, it is possible that elderly individuals who are already predisposed to gastrointestinal ailments might be more sensitive to the negative effects of DON.

One potential cause of DON-induced anorexia is the induction of proinflammatory cytokines, including IL-1β, IL-6, and TNF-α, which have been previously shown to cause sickness behavior in humans and experimental animals [17,18,19]. Our lab has reported the induction of these cytokines in female mice, as well as an increased plasma IL-6 response in male mice in comparison with female mice [5,20,21]. Prior investigations of the effect of lipopolysaccharide (LPS) in aged and adult mice have shown that the former exhibit a greater cytokine response to LPS than adults [22,23]. Huang *et al.* (2008) found that plasma IL-6 levels of aged mice were 2- and 6.6-fold higher at 2 and 8 h post intracerebroventricular (i.c.v.) injection of 10 ng of LPS than adult mice [22]. Another study found that plasma levels of IL-1β and IL-6 were significantly increased in aged mice in comparison with adults at 6 h post exposure to 5 μg/g bw LPS administered by intraperitoneal (i.p.) injection [23].

Additionally, our lab discovered that the gut satiety hormones peptide YY (PYY) and cholecystokinin (CCK) are induced in mice upon i.p. and oral DON exposure and both contribute to feed refusal caused by this mycotoxin [24,25]. When Stanström *et al.* (1998) compared the number of enteroendocrine cells responsible for PYY secretion in the intestinal epithelium of mice that were 3 and 24 months of age, they found that aged mice had significantly more of these cells [26]. Another study in rats revealed that PYY-containing cells per colonic crypt increased with age [27]. In a study comparing the effects of CCK in adult and aged mice, researchers found that aged mice showed increased sensitivity to CCK and that the action of CCK was prolonged in aged mice [28]. As PYY and CCK are induced by DON and tend to increase with age, exploring the combined effects of DON exposure and aging is of importance.

The aims of this study were to compare feed refusal responses between adult and aged mice after acute and dietary DON exposure. Study 1 compared feed refusal differences by age following acute i.p. DON exposure. In Study 2, we compared DON tissue concentrations, proinflammatory plasma cytokine responses, and satiety hormone responses in adult and aged animals following acute i.p. exposure to the toxin. Finally, Study 3 compared differences in food intake and body weight suppression upon dietary DON exposure in adult and aged mice. The results presented herein indicate that in aged mice the food refusal response is greater than in adult mice following both acute i.p. and dietary DON exposure and that acute exposure is accompanied by delayed DON tissue clearance as well as increased proinflammatory cytokine and gut satiety hormone responses in aged mice.

## 2. Results

### 2.1. Study 1

#### DON-Induced Feed Refusal Is Greater in Aged Mice than in Adult Mice

**Figure 1:**
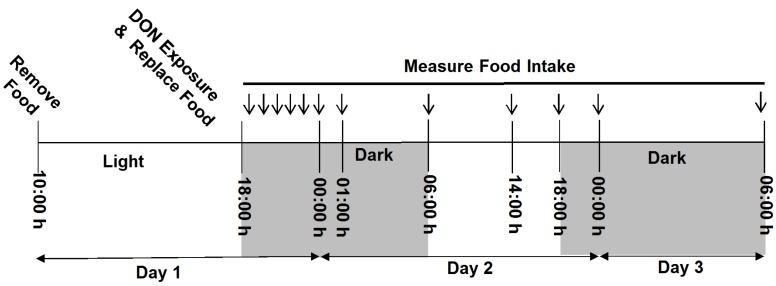
Study 1 experimental design. On day 1, mice were fasted from 10:00 to 18:00 h, then exposed to DON treatments or vehicle control. Food was immediately replaced following exposure and food measurements were recorded hourly from 1 to 7 h PI, and at 12, 20, 24, and 36 h PI as indicated by arrows.

**Figure 2:**
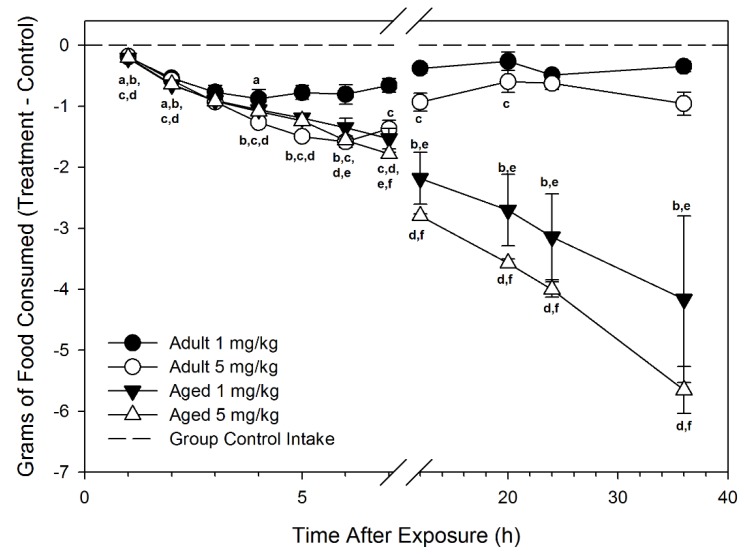
Aged mice are more sensitive to DON-induced feed refusal than adults following acute i.p. exposure. Food intake is cumulative at the time point and was normalized to group control average. Data are mean ± SEM (*n* = 5/gp). Significant differences (*p* < 0.05) are indicated as follows: a = 1 mg/kg bw DON-dosed adult different from adult control; b = 1 mg/kg bw DON-dosed aged different from aged control; c = 5 mg/kg bw DON dosed adult different from adult control; d = 5 mg/kg bw DON-dosed aged different from aged control; e = aged different from adult at 1 mg/kg bw DON; and f = aged different from adult at 5 mg/kg bw DON.

The effects of acute i.p. exposure to 1 mg/kg and 5 mg/kg bw DON were compared in aged and adult mice over 36 h (Figure 1). Aged mice treated with 1 mg/kg DON consumed less food than control aged mice from 1 h to 36 h post-injection (PI) (Figure 2). In comparison, adult mice treated with the same dose of DON consumed less food than adult controls at 1 to 4 h PI and then began to show recovery in food intake. By 6 h PI, aged mice exposed to 1 mg/kg DON ingested significantly less food than adult mice. At this dose, aged mice consumed 82% less food than aged control mice, while adult mice at this dose had consumed 41% less food than adult controls at this time point.

Following exposure to 5 mg/kg bw DON, total food consumption was reduced from 1 to 20 h PI and 1 to 36 h PI for adult and aged mice, respectively. At 7 h PI, 5 mg/kg bw DON-treated adult mice consumed 32% feed of adult vehicle controls, whereas DON-treated aged mice ate only 2% of that of aged vehicle controls.

At 36 h PI, aged mice continued to exhibit a substantial depression in food intake compared to aged control mice, with food consumption accounting for 43% and 22% of that of control aged mice at 1 mg/kg and 5 mg/kg bw DON, respectively. In comparison, food intake in adult mice treated with 1 mg/kg and 5 mg/kg bw DON at 36 h PI was 94% and 84%, respectively, of adult control food intake, suggesting that this group has nearly fully recovered from the toxin.

### 2.2. Study 2

#### 2.2.1. DON Tissue Concentrations Are Higher in Aged Mice Compared with Adults after Acute Exposure

**Figure 3:**
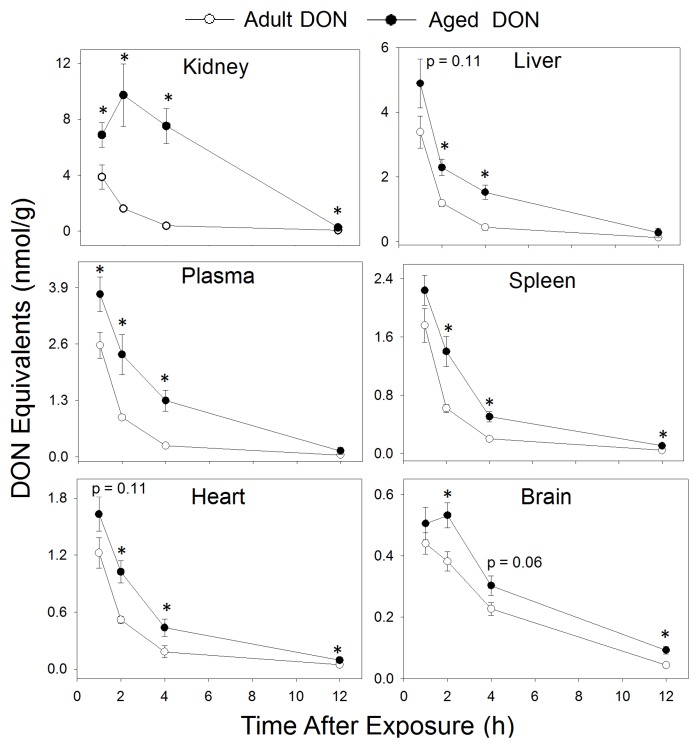
DON equivalent concentrations are higher in aged mice than adult mice post-acute i.p. exposure to 1 mg/kg bw DON. DON is reported as DON equivalents because the Veratox HS ELISA was fully cross reactive with DON3GlcA. Control animals did not have detectable concentrations of DON (data not shown). Data are mean ± SEM (*n* = 8–10/gp). Asterisk indicates statistical significance from adults at time point (*p* < 0.05).

When DON tissue concentrations in the kidney, liver, plasma, spleen, heart, and brain were assessed over 12 h following i.p. exposure to DON at 1 mg/kg bw, toxin levels were consistently higher in aged mice than in adult mice (Figure 3). The initial rank order of DON concentrations in both aged and adult mice was: kidney > liver > plasma > spleen > heart > brain. At 1 h PI, aged mice had higher DON concentrations in the kidney (1.8-fold increase) and plasma (1.5-fold increase). At 2 h PI, DON concentrations were higher in all tissues of aged mice compared with adults, ranging from 6-fold higher in the kidney to 1.5-fold higher in the brain. Interestingly, DON concentrations in kidneys and brains of aged animals were higher at 2 h PI than at 1 h PI. At 4 h PI, DON concentrations were significantly higher in all organs of aged mice except the brain, with the largest differences existing in the kidney (20-fold higher) and plasma (5-fold higher). Aged mice continued to exhibit higher DON levels in the kidney, spleen, heart, and brain at 12 h PI, with concentrations ranging from 4.3 to 2-fold above the levels of adult mice.

#### 2.2.2. Plasma IL-6 and IL-1β Are Higher in Aged Mice Compared with Adults after Acute DON Exposure

**Figure 4:**
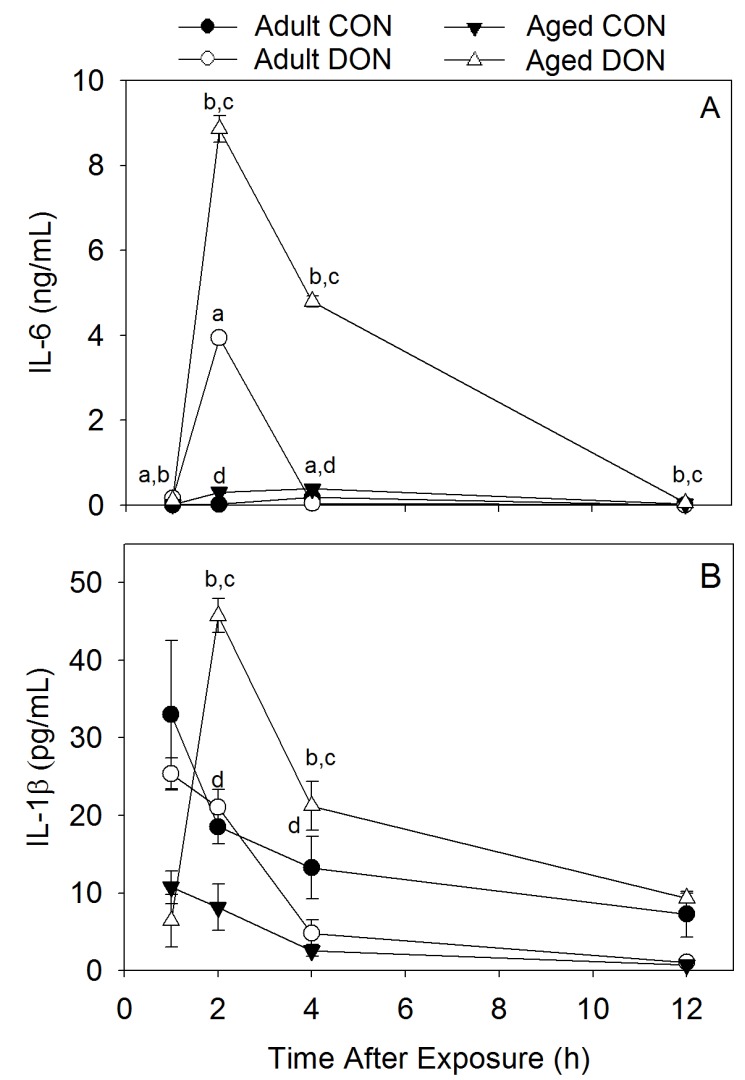
Aged mice exhibit higher IL-6 and IL-1β plasma levels post-acute i.p. exposure to 1 mg/kg bw DON. (**A**) IL-6 plasma levels ng/mL and (**B**) IL-1β plasma levels pg/mL. Data are mean ± SEM (*n* = 4/replicates). Significant differences are indicated as follows: a = DON-dosed adults different from adult control; b = DON-dosed aged different from aged control; and c = DON-dosed aged different from DON-dosed adult (*p* < 0.05); d = vehicle control age groups different.

When the effects of acute exposure to 1 mg/kg bw DON on plasma proinflammatory cytokines were assessed, aged mice exhibited higher plasma levels of IL-6 and IL-1β than adult animals (Figure 4). Beginning at 2 h PI, aged mice treated with DON had a 2.2-fold increase in both plasma IL-6 and IL-1β when compared with adult mice at 2 h PI. At 4 h PI, aged mice had a 120-fold difference of IL-6 in comparison with adult animals, which had nearly returned to adult control levels at this time point. Aged animals also had a 4.4-fold increase in IL-1β at 4 h PI when compared with adult mice. Plasma IL-6 levels remained significantly elevated in aged mice when compared with control aged mice and DON-treated adult mice at 12 h PI, while IL-1β levels were not statistically significant in any group at this time point. Vehicle-treated aged mice had higher plasma IL-6 at 2 and 4 h post exposure than treated adults. However, IL-6 levels were very low compared to DON-treated mice at these time points. Adult mice had higher levels of IL-1β at 2 and 4 h PI. TNF-α was not detectable in the plasma of either aged or adult mice at any time point. This latter result is in agreement with a previous study in our lab comparing proinflammatory cytokines in male and female mice treated with 1 mg/kg bw DON [21].

#### 2.2.3. DON Induction of PYY and CCK Is Greater in Aged Animals than in Adults with DON Exposure

**Figure 5:**
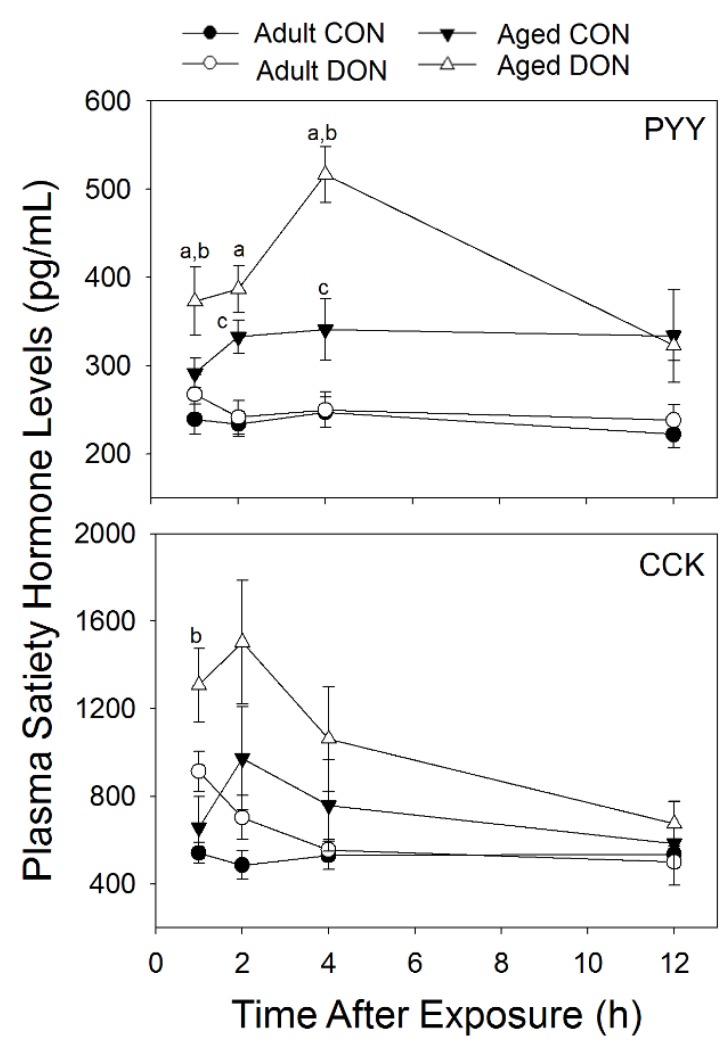
DON induction of plasma PYY and CKK is greater in aged mice after acute i.p. exposure. Data are mean ± SEM (*n* = 8–10/gp). Significant differences (*p* < 0.05) are indicated as follows: a = DON-dosed aged different from DON-dosed adult; b = DON dosed aged different from aged control; and c = control aged different from control adult.

When the effects of acute exposure to 1 mg/kg bw DON on plasma levels of gut satiety hormones were measured, DON elicited greater increases in PYY and CCK in aged mice than in adult mice (Figure 5). DON-treated aged mice exhibited a trend toward greater PYY than similarly treated adult mice at 1 and 2 h PI. At 4 h PI, aged mice treated with DON exhibited a robust increase in plasma PYY, having levels that were 51% higher than control aged mice. In contrast, adult mice treated with DON at this time point were not different from control adults. Plasma PYY had returned to baseline levels by 12 h PI in both DON-treated groups. In mice exposed to the vehicle only, PYY plasma levels trended higher in aged mice than adults and PYY was significantly higher in control aged mice at 2 and 4 h PI than in control adults.

DON-treated aged mice exhibited a trend toward greater CCK than similarly treated adult mice at 1 and 4 h PI. At 1 h PI, aged mice exposed to DON had a 99% increase in plasma CCK in comparison to control aged animals and were significantly higher than control aged mice and DON-treated adults. In comparison, while adult mice exhibited a 59% increase in CCK over adult controls at 1 h PI, they were not significantly higher than control adults. DON-treated animals continued to show trends toward increased plasma levels of CCK at 2 and 4 h PI, although these were not statistically significant. At 12 h PI, CCK concentrations were no longer impacted by DON in either age group. CCK plasma levels were higher in vehicle-treated aged mice than in adults, though not significantly.

### 2.3. Study 3

#### 2.3.1. Reduction in Food Intake Is Greater in Aged Mice than in Adults with Dietary DON Exposure

The impact of dietary exposure to DON at 0, 1, 2.5, and 10 ppm on food intake was compared between aged and adult mice. Aged mice showed a greater reduction in food intake upon dietary DON exposure than adult mice (Table 1). After 1 day of dietary DON exposure, both aged and adult animals exhibited a statistically significant negative correlation between decreasing food intake with increasing DON concentration in the diet. Aged mice fed DON diets containing 2.5 and 10 ppm consumed 9% and 62% less food, respectively, than aged control mice over the first day, while adult mice fed the same concentration diets consumed 2% and 58% less food than adult controls, respectively, at this time point. After 2 days, aged mice continued to exhibit decreasing food intake with increasing DON concentration, while adult mice no longer exhibited this negative correlation. Aged mice fed diets containing 2.5 and 10 ppm DON consumed 13% and 54% less food, respectively, than aged control mice at day 2, while adult mice fed the same concentration diets consumed 3% and 10% less food than adult controls at this time point.

**Table 1:** Reduction in food consumption is greater in aged mice fed a DON-containing diet. Values are percent of group control food intake. Data are mean ± SEM (*n* = 4–5/gp).

Age Group	Conc. DON Diet	% Control Food Intake 0–24 h	Correlation Coefficients	% Control Food Intake 24–48 h	Correlation Coefficients
Adult	1 ppm	103.8 ± 9.1	*r* = −0.848	97.8 ± 7.3	*r* = −0.275
Adult	2.5 ppm	98.3 ± 8.1		96.7 ± 5.1	
			*p* = 0.000002		*p* = 0.274
Adult	10 ppm	42.4 ± 6.9		90.4 ± 7.3	
Aged	1 ppm	101.0 ± 5.9	*r* = 0.810	92.7 ± 7.3	*r* = −0.775
Aged	2.5 ppm	90.5 ± 13.5		87.2 ± 8.1	
			*p* = 0.00003		*p* = 0.0001
Aged	10 ppm	37.8 ± 5.2		45.9 ± 10.6	

#### 2.3.2. Dietary DON Transiently Suppresses Body Weights to a Greater Extent in Aged Mice

**Figure 6:**
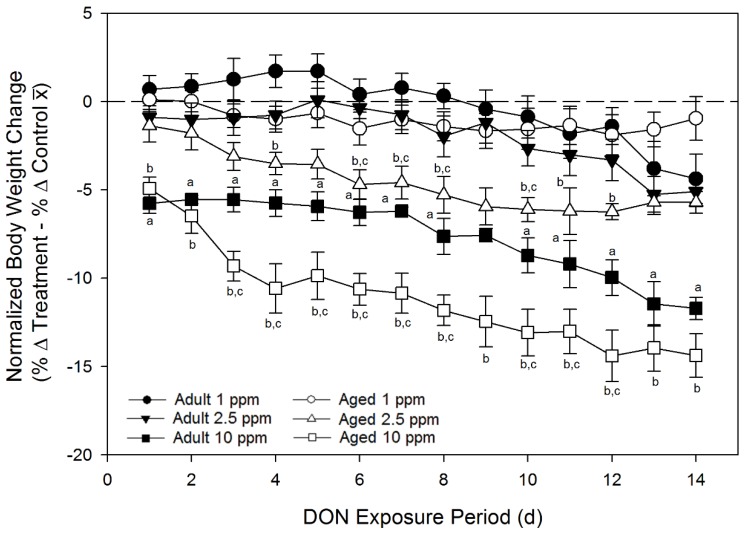
Aged mice are more sensitive to DON-induced anorexia than adult mice upon dietary DON exposure. Changes in daily body weights were determined from weight at study initiation and normalized to group control weight gain. Dashed line indicates group control body weight. Data are mean ± SEM (*n* = 8–10/gp). Statistical differences (*p* < 0.05) are indicated as follows: a = DON-treated adult different from control adult; b = DON-treated aged different from control aged; and c = DON-treated aged different from DON-treated adult fed same ppm DON diet.

Effects of consuming feed containing 1, 2.5, and 10 ppm DON on body weight suppression were further compared by age. Aged mice were more sensitive to body weight suppression than adults (Figure 6). Aged mice fed a diet containing 2.5 ppm DON weighed less than group control mice and adult mice fed the same diet beginning on day 6 of the exposure period, weighing 5% less than aged control mice, while adult treated mice weighed 0.4% less than adult control mice. At the study termination (day 14), aged mice fed 2.5 ppm DON weighed 6% less than aged control mice. Adult mice fed the same diet weighed 5% less than adult controls at day 14 and the significance between the differences in weight loss by age no longer existed. Aged mice fed a diet containing 10 ppm initially exhibited a rapid depression of weight gain in comparison with controls and on day 4 weighed 11% less than aged control mice. In comparison, adult mice fed 10 ppm DON diet weighed 6% less than adult controls at day 4. After 14 days of exposure to a diet containing 10 ppm DON, aged and adult mice continued to show significant weight suppression in comparison to the respective group controls; however, differences between adult and aged mice were no longer evident upon termination of the study, suggesting that these effects were transient.

#### 2.3.3. Tissue Concentrations of DON Equivalents (DON + DON3GlcA) Are Elevated Dose Dependently with Dietary Exposure in Aged Mice More than Adults

DON equivalent tissue concentrations were compared between aged and adult mice after 14 days of dietary DON exposure. Tissue concentrations elevated with increasing DON exposure; however, the increase was only significant in mice fed diets containing 10 ppm DON, with the exception of the DON levels being significantly higher in the spleens of mice fed a diet containing 2.5 ppm DON (Supplemental Materials, Figure S1). No significant differences in DON equivalent tissue concentrations were observed between aged and adult mice; however, adult mice fed diets containing 10 ppm DON had higher DON equivalent concentrations in the spleen than aged animals fed the same diet. As animals were allowed *ad libitum* access to food prior to euthanasia, the amount of toxin consumed and time of consumption are unknown. The observation that aged mice tended to have lower DON equivalent levels than adult mice fed the same diet could indicate that aged animals were consuming less food, and thus less toxin, than adults.

#### 2.3.4. Proinflammatory Plasma Cytokines Are not Detectable after 14 Days of Dietary DON Exposure

Plasma levels of the proinflammatory cytokines IL-6, IL-1β, and TNF-α were not detectable in aged or adult mice after 14 days of dietary DON exposure at any treatment level. These findings are consistent with previous research in our lab also reporting no change in the plasma levels of IL-6, IL-1β, or TNF-α after 17 days of exposure to diets containing 1, 2.5, and 10 ppm DON in either male or female mice [21]. A study examining mice exposed to 2 mg/mL DON in drinking water for 36 days could not detect these proinflammatory cytokines in the plasma [29].

## 3. Discussion

Prior investigations have addressed increased susceptibility of young mice, poultry, and swine to DON, but the negative effects experienced by older animals has been largely unstudied [5,6,7]. This study is the first to report increased anorectic responses in aged mice compared with young adults following DON exposure. The discovery that anorectic responses to DON are greater in old animals is critical because advanced age in humans leads to a phenomenon known as the “anorexia of aging” in which elderly individuals show unintended weight loss [9,12,13]. There is a possibility that DON consumption could exacerbate this condition.

The finding that aged mice have higher tissue concentrations of DON following acute exposure over a prolonged period compared with adults suggests that aged mice might differ in their ability to absorb, metabolize, or excrete the toxin. The contention that aged animals have a lower capacity to metabolize and thus excrete DON is supported by the previous observation of lower mRNA levels of UDP-glucuronosyltransferases, the family of enzymes responsible for DON glucuronidation, in aged mice when compared with their adult counterparts [30].

Increased levels of DON in the kidneys of aged mice relative to adults are consistent with the notion that aged animals have a reduced capacity to excrete the toxin. As kidney DON concentrations in aged mice were higher at 2 h PI than at 1 h PI, it appears that the toxin accumulated in the kidney. This result could be due to changes in metabolic abilities or to reduced kidney functioning, including reduced glomerular filtration rate and renal blood flow, known pharmacokinetic changes observed with aging [15,31]. Analyzing differences in the DON metabolite profile in different tissues and the integrity of kidney functioning with aging will therefore be an important objective in future studies.

DON concentrations in the brains of aged mice were also elevated at 2 h PI compared with 1 h PI, as seen in the kidney, while the levels of this toxin in the brains of young adults decreased from 1 h PI to 2 h PI. Importantly, DON concentrations were still higher in aged mice at 12 h PI. These findings are notable because DON is known to directly affect the brain, including the induction of c-Fos, a strong indicator of neuronal activation [19,32,33]. Further exploration of the possible differential effects of DON on the brains of aged mice could provide new insight into why animals are more sensitive than adults to the anorectic effects of DON.

Increased proinflammatory plasma cytokine responses in aged mice compared with adults might be a critical factor behind the heightened anorectic responses observed in aged mice [34]. Previous investigations of LPS-induced cytokine responses also reported an increase in proinflammatory cytokine responses in aged animals compared with adults [22,23]. Here, we observed that peak elevation of plasma IL-1β and IL-6 at 2 h PI in aged mice coincides with the highest kidney concentration of DON. Together these observations suggest that a decreased ability to excrete DON is linked to increased proinflammatory cytokine responses. Elevated tissue IL-1β might be more important to DON-induced anorexia than the observed IL-6 increase for several reasons. First, a recent study by Wu and Zhang [35] reported that type 1 IL-1 receptor (IL-1R1) antagonist IL-1RA dose-dependently attenuated both IL-1 beta- and acute DON-induced anorexia. Second, we have previously observed that IL-6 deficient mice are recalcitrant to food refusal caused by dietary DON exposure [36]. Third, while both peripheral and central injection of IL-1β into rodents elicits anorexia in rodents, only central injection of IL-6 suppresses food intake [34]. Thus, the contributions of DON-induced IL-6 to food refusal are unclear at this time and require further study, particularly with regard to the effects of this trichothecene on the expression of IL-6 in the brain.

Some studies have reported increased baseline levels of proinflammatory cytokines in aged mice, a process known as inflammaging [37,38]. Plasma IL-6 was slightly elevated in aged control mice (0.02–0.3 ng/mL) in our studies of acute i.p. exposure in comparison with adults (0.01–0.02 ng/mL). The baseline levels of plasma IL-6 are likely a response to the i.p. injection. As no elevation in plasma cytokine levels was observed at 12 h PI in control aged mice, elevated plasma cytokine levels in DON-treated aged mice are a result of toxin exposure and not a proinflammatory phenotype prior to exposure, and highly consistent with their potential to contribute to the increased anorectic response in aged mice.

Elevated PYY and CCK responses in aged mice relative to adults could also elicit increased food refusal in aged mice. These hormones are known to cause satiety and have been previously shown by our lab to increase with exposure to DON [24,25,39]. Again, increased PYY and CCK could be caused by slower excretion of DON. It is also possible that DON evokes a greater induction of these gut satiety hormones innately, as previous research in humans has shown greater induction of PYY and CCK with food consumption in aged subjects compared with younger individuals [40,41,42]. Our findings also support that PYY, and to a lesser extent CCK, are indeed basally elevated in aged animals and exposure to DON elicited a further elevation of these satiety hormones. One limitation of the acute studies is that food intake and hormones were measured upon acute i.p. exposure to DON to minimize stress to aged mice and microflora effects. Future research should also address whether these satiety hormones are similarly elevated in aged mice with oral exposure to DON.

The greater suppression of food intake in aged mice than in adults upon dietary DON exposure is consistent with the observation of greater feed refusal in aged animals *vs.* adults in the acute i.p. exposure to DON. Aged mice consuming less food than adult animals also indicates that they are more sensitive to the adverse effects of DON. Because aged animals ingest less food, they also consume less DON, suggesting that aged mice have a lower threshold for the amount of toxin that they can tolerate.

At the termination of Study 3, weight loss in aged and adult mice appeared to plateau. It might be speculated that this results from induction by DON of UGT enzymes and/or bacterial ability to convert DON to DOM-1, resulting in a greater capacity to metabolize DON. In a two-year study assessing chronic exposure of mice to diets containing 1, 5, and 10 ppm DON, body weight gain appeared to be unaffected by DON exposure for the first 100 days of treatment diets [43]. After this time point, weight gain in mice on diets containing 5 and 10 ppm begins to decrease while the body weight gain of mice on diets containing diets 0 and 1 ppm DON continued to increase until approximately 500 days of treatment diets. The results presented in the two-year chronic feeding study, along with our findings in Study 3, indicate that while some adaptation to DON does occur, chronic exposure to DON over a lifetime will still result in weight reduction.

## 4. Experimental Section

### 4.1. Animals

The Institutional Animal Care and Use Committee at Michigan State University approved all animal experiments. Adult male (3 mos) and aged male C57BL6 mice (22 mos) were purchased from the National Institute on Aging colony (Charles River Laboratories, Wilmington, MA, USA). Mice were housed singly in polycarbonate cages with sifted aspen bedding on a 12 h light/dark cycle, with constant temperature (21–24°C) and humidity (40%–55%). In all experiments mice were acclimated to a high fat pellet diet (45% kcal from fat; Research Diets, Inc., New Brunswick, NJ, USA) one week prior to DON exposure. The high fat diet has previously been determined to provide the most efficient food recovery in acute DON exposure studies [44].

### 4.2. DON

DON used in i.p. injections and experimental diet was obtained from Dr. Tony Durst (University of Ottawa, Ottawa, ON, Canada) and purity was verified to be 98% by elemental analysis (Galbraith Labs, Knoxville, TN, USA). For all i.p. injections, DON was dissolved in sterile Dulbecco’s phosphate buffered saline (PBS; Sigma-Aldrich, St. Louis, MO, USA) to yield 100 µL injection volumes. High fat pellet diets containing 0, 1, 2.5, and 10 ppm DON were formulated by Research Diets, Inc. (New Brunswick, NJ, USA); the DON concentration of diets was confirmed using the Veratox high sensitivity (HS) enzyme-linked immunosorbent assay (ELISA; Neogen, Lansing, MI, USA) according to the manufacturer’s protocols.

### 4.3. Experimental Design

#### 4.3.1. Study 1

Effects of age on food intake after acute i.p. DON exposure were assessed as summarized in Figure 1 using a protocol previously described by our laboratory [44]. After acclimation, adult and aged mice (*n* = 5/group) were fasted from 10:00 to 18:00 h, and injected i.p. with either 0 (vehicle), 1 or 5 mg/kg bw DON. Food consumption was measured hourly 1 to 7 h post exposure, and at 12, 20, 24, and 36 h post injection (PI). Measurements were conducted under red light conditions during dark cycle. We choose to use i.p. exposure for this and the following study to (1) minimize stress [45] and (2) bypass microbial metabolism to DOM-1, a non-toxic metabolite of DON, because rodent gastrointestinal tract microflora are capable of forming this metabolite, while human microflora are typically unable of this type of metabolism [46].

#### 4.3.2. Study 2

Effects of age on DON tissue levels and plasma concentration of proinflammatory cytokines and satiety hormones were measured after acute i.p toxin injection. Adult and aged mice (*n* = 9–10/group) were fasted as in Study 1 and exposed to 1 mg/kg bw DON in PBS or PBS vehicle via i.p. injection. Mice were euthanized with CO_2_ at 1, 2, 4, and 12 h PI without food replacement. Blood was collected via cardiac puncture and then kidney, liver, spleen, heart, and brain samples were collected. Plasma was isolated from blood by centrifugation at 3500× *g* for 10 min at 4 °C. Plasma and organs were stored at −80 °C until analysis.

#### 4.3.3. Study 3

Effects of age on food intake and body weight changes elicited by dietary DON exposure were assessed. After acclimation, adult and aged mice were randomized into equal weight groups by age group (*n* = 5–6/group) and placed on high fat diets containing 0, 1, 2.5, and 10 ppm DON. Mice were given two weighed pellets (approximately 7 g) to allow for *ad libitum* food consumption. Body weights were measured daily at 10:00 AM for 14 days. Food intake was also measured each day at this time for 2 days. After 2 days, mice on diets containing DON progressively began to shred the pellets into fine particles, preventing accurate food recovery. After 14 days of DON exposure, mice were euthanized under CO_2_ at 8:00 AM. Food access was *ad libitum* prior to euthanasia. Blood was collected via cardiac puncture and then kidney, liver, spleen, heart, and brain samples were collected. Plasma was isolated from the blood. Plasma and organs were stored at −80 °C until analysis.

### 4.4. Analytical

#### 4.4.1. DON Analysis

DON quantification in plasma and organs in Study 2 were analyzed using the Veratox high sensitivity (HS) ELISA (Neogen, Lansing, MI, USA) as described by Pestka *et al.* (2008), with slight modifications. Briefly, organs were homogenized 1:1 in PBS (except the heart, which was homogenized 1:2). Tissue homogenates were heated at 100 °C for 5 min and then centrifuged at 14,000× *g* for 10 min at 4 °C. The resulting supernatant was analyzed by ELISA. Chromogenic end product was measured using an F3 ELISA plate reader at 650 nm and Softmax software (Molecular Devices, Menlo Park, CA, USA). As previously described by our lab [21], the ELISA was found to be 100% cross reactive with DON3GlcA, a major metabolite of the toxin produced in rodents. Cross reactivity with other potential glucuronides was not determined. Since we did not measure ratio of active *vs*. conjugated DON, results are reported as DON equivalents.

#### 4.4.2. Proinflammatory Cytokine Analyses

Plasma levels of the proinflammatory cytokines IL-6, IL-1β, and TNF-α were determined in Study 2 and 3 using Duoset ELISAs from R & D Systems (Minneapolis, MN, USA). Due to limited plasma quantities, equal volumes of mouse plasma samples from Study 2 were pooled within groups and ran in technical replicates (*n* = 4 rep).

#### 4.4.3. Satiety Hormone Analyses

Plasma levels of the gut satiety hormones CCK and PYY3-36 were analyzed using ELISA kits for CCK (CCK [26,27,28,29,30,31,32,33], nonsulfated; human-, rat-, and mouse-specific) and PYY (PYY [3,4,5,6,7,8,9,10,11,12,13,14,15,16,17,18,19,20,21,22,23,24,25,26,27,28,29,30,31,32,33,34,35,36]; mouse-, rat-, porcine-, and canine-specific; Phoenix Pharmaceuticals, Burlingame, CA, USA) in with plasma samples from Study 2. CCK and PYY were not measured in Study 3 because animals had *ad libitum* access to food prior to study termination.

### 4.5. Statistical Analysis

Statistical analysis was conducted by SigmaPlot version 11.0 (Jandel Scientific; San Rafael, CA, USA). Statistical comparisons between ages were made at each time point using a Student’s *t*-test, unless normality failed. If normality was not met, a Mann-Whitney Rank Sum test was performed. Statistical comparisons between age and dose were made at each time point using a one-way analysis of variance (ANOVA), unless normality failed. A Kruskal–Wallis one-way analysis of variance by ranks was performed if normality was not met. Student–Neuman–Keuls was used in all *post hoc* analysis for parametric and non-parametric animal groups of equal numbers. Dunn’s test was used in *post hoc* analysis of non-parametric analysis of unequal animal group numbers. Pearson product moment correlations were performed to determine the statistical significance between food consumption and treatment levels of DON diets by age and day in Study 3. Differences were considered significant when *p* < 0.05.

## 5. Conclusions

The results presented herein indicate that aged mice are more susceptible than their young adult counterparts to DON-induced anorexia following either acute i.p. or dietary DON exposure. In addition to reduced food intake in dietary exposure studies, a greater suppression of body weight gain was seen in aged mice compared with adult mice. Aged mice exhibited slower tissue clearance of DON and increased proinflammatory plasma cytokine and gut satiety hormone responses compared with adult mice after acute i.p. exposure to DON. Collectively, these findings suggest that advanced life stage should be considered when formulating risk assessments for DON and other trichothecene mycotoxins.

## Supplementary Materials

Supplementary materials can be accessed at: http://www.mdpi.com/2072-6651/7/10/4199/s1.
