# Supplementary material for: High Sensitivity of Aged Mice to Deoxynivalenol (Vomitoxin)-Induced Anorexia Corresponds to Elevated Proinflammatory Cytokine and Satiety Hormone Responses
Source: Toxins (Basel). 2015 Oct 19;7(10):4199–215. doi: 10.3390/toxins7104199 (PMC4626729; doi:10.3390/toxins7104199)
Supplement: Supplementary file 1 [file toxins-07-04199-s001.pdf]

Supplementary Materials

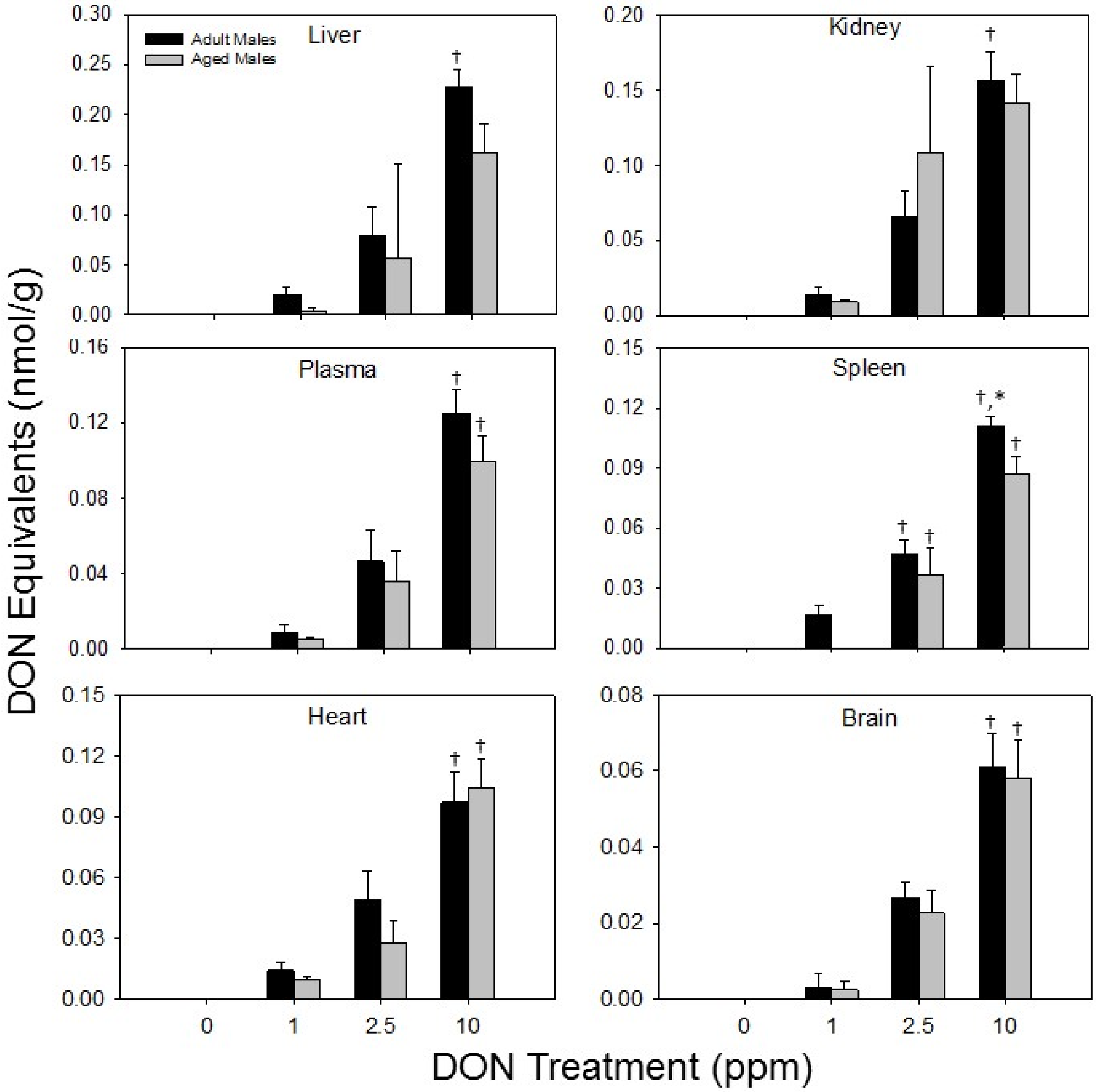

Figure S1. Tissue DON concentrations increase in a dose dependently after dietary DON exposure in mice. Data are mean ± SEM (n = 6/gp). Asterisk indicates statistical significance from aged mice on same treatment diet and dagger indicates significance from control diet within age group ($p < 0.05$).
